# Butterfly Garden Connects Community Living Center Residents, Family, and Staff During the COVID-19 Pandemic

**DOI:** 10.1093/geroni/igab046.3564

**Published:** 2021-12-17

**Authors:** Maribel Rodriguez-Gonzalez, Maura Miller, Gelda Pratt, Micheal A Silverman, Sandra DiScala

**Affiliations:** 1 Florida Atlantic University- West Palm Beach VA Medical Center, West Palm Beach VA Medical Center/West Palm Beach, Florida, United States; 2 West Palm Beach VA Medical, West Palm Beach VA Medical Center/ West Palm Beach, Florida, United States; 3 West Palm Beach VA Medical Center, West Palm Beach VA Medical Center, Florida, United States; 4 West Palm Beach VA Medical Center, West Palm Beach VA Medical Center/ Geriatric Extended Care, Florida, United States; 5 West Palm Beach VA Medical Center, West Palm Beach VA Medical Center/West Palm Beach, Florida, United States

## Abstract

The COVID-19 Pandemic has led to significant morbidity and mortality in older residents of long-term care facilities. In addition, the stringent restrictions on visitation of family and loved ones has further socially isolated residents leading to an increase in depression, loneliness, and spiritual distress. The Community Living Center (CLC) staff at West Palm Beach VA Medical Center wanted to address this dilemma and created a unique “Butterfly Garden” (BG) visitation space. This space is a therapeutic garden adjacent to the CLC that can be accessed by families without having to enter the facility. Participants in the BG reported feelings of peace, undisturbed reflection, and tranquility as they observed and experienced nature’s life cycle. This show of nature’s beauty promotes visual, tactile, and olfactory sensory stimulation while attracting hummingbirds, bees, butterflies, and peace to this calm garden space. The BG visitations offers residents, family, and staff opportunities to experience the health benefits of nature during their visits under strict CDC social contact guidelines. From September through December 2020 and from January through March 2021 there were 67 and 184 visits respectively as families became more involved. The feedback from residents, families, and staff indicated that the spirits of all participants were raised by the BG visits despite the difficult challenges of social distancing and limited CLC visitations. This BG concept could serve as a model for other long-term care facilities to allow socially distant family visits to loved ones in a safe nature-based environment of care with or without a pandemic.

